# Multiparametric MRI‐Based Intratumoral and Peritumoral Radiomics for Distinguishing Solitary Intrahepatic Mass‐Forming Cholangiocarcinoma From Colorectal Liver Metastases

**DOI:** 10.1002/cam4.71120

**Published:** 2025-08-05

**Authors:** Liyong Zhuo, Xiaomeng Li, Shuo Dai, Lihong Xing, Zijun Song, Xueyan Liu, Jianing Wang, Caiying Li, Xiaoping Yin

**Affiliations:** ^1^ Department of Radiology Affiliated Hospital of Hebei University Baoding People's Republic of China; ^2^ Department of Mathematical Sciences Liaocheng University Liaocheng Shandong People's Republic of China; ^3^ Department of Critical Care Medicine Baoding First Central Hospital Baoding People's Republic of China; ^4^ Department of Medical Imaging The Second Hospital of Hebei Medical University Shijiazhuang People's Republic of China

**Keywords:** cholangiocarcinoma, colorectal liver metastases, differential diagnosis, MRI, radiomics

## Abstract

**Objective:**

To establish a model based on intratumoral and peritumoral radiomics for preoperatively differentiating solitary intrahepatic mass‐forming cholangiocarcinoma (IMCC) lesions from colorectal cancer liver metastases (CRLM).

**Methods:**

Preoperative MRI scans from IMCC patients were retrospectively obtained from three academic medical centers. Radiomics features were extracted from the intratumoral and multiple peritumoral regions. After feature selection, the optimal peritumoral range was determined. The radiomics model was developed by integrating both single‐sequence and multisequence models through probabilistic ensemble learning. Significant variables from clinical imaging features, radiomics, were integrated into a combined model, and its performance was evaluated using the area under the receiver operating characteristic curve (AUC).

**Results:**

A total of 170 patients (93 IMCC, 77 CRLM) comprised the training cohort, and 42 (23 IMCC, 19 CRLM) formed the external validation cohort. The Combined model achieved superior AUCs in training (0.978 [95% CI: 0.971–0.985]) and validation (0.940 [0.899–0.968]), outperforming radiomics (training: 0.947 [(95% CI: 0.932–0.961]); validation: 0.908 [(95% CI: 0.861–0.943)]) and Clin‐Imag models (training: 0.858 [95% CI: 0.831–0.880]; validation: 0.842 [95% CI: 0.786–0.889]) (DeLong test, *p* = 0.001). SHAP analysis identified DWI‐based 5‐mm peritumoral features and clinical‐imaging variables (e.g., lesion location and bile duct dilation) as key discriminators.

**Conclusions:**

The combined model integrating clinical‐imaging variables and multiparametric MRI‐derived intratumoral and 5‐mm peritumoral radiomics features provides a non‐invasive tool for distinguishing solitary IMCC from CRLM, offering potential clinical utility for guiding personalized treatment strategies and avoiding unnecessary invasive interventions.

**Abbreviations**
AUC: area under the receiver operating characteristic curve
CI: confidence interval
CRLM: colorectal cancer liver metastases
DWI: diffusion‐weighted imaging
GLCM: gray‐level co‐occurrence matrix
GLDM: gray‐level dependence matrix
GLRLM: gray‐level run length matrix
GLSZM: gray‐level size zone matrix
HBP: hepatobiliary phase imaging
ICC: intrahepatic cholangiocarcinoma
IMCC: intrahepatic mass‐forming cholangiocarcinoma
LASSO: least‐absolute shrinkage and selection operator
MRI: magnetic resonance imaging
NGTDM: neighboring gray‐tone difference matrix
NPV: negative predictive value
PPV: positive predictive value
RFE: recursive feature elimination
ROC: receiver operating characteristic
SHAP: Shapley additive explanations
T2WI: T2‐weighted imaging
VOI: volume of interest

## 1 Introduction

Distinguishing single intrahepatic mass‐forming cholangiocarcinoma (IMCC) from solitary colorectal liver metastases (CRLM) represents an urgent clinical challenge with profound therapeutic implications. IMCC is the second most common primary intrahepatic cholangiocarcinoma (ICC) [1], and CRLM is the predominant metastatic liver tumor from colorectal cancer [1, 2], exhibiting striking overlaps across imaging modalities and histopathology. On multiparametric MRI, both entities share similar morphological features and signal characteristics: heterogeneous arterial enhancement, hypointensity in portal/delayed phases [3, 4], and overlapping DWI/T2W patterns [5, 6]. Histologically, the coexistence of adenocarcinoma patterns, fibrotic stroma, and necrotic components further blurs diagnostic boundaries [7]. These ambiguities carry critical consequences, as up to 25% of presumed IMCC cases ultimately prove to be metastatic disease at surgical histology, often necessitating additional invasive colorectal evaluations [8]. Given divergent treatment paradigms where IMCC requires radical hepatic resection versus CRLM demanding combined colorectal and liver interventions [9], an accurate preoperative differentiation strategy is imperative to optimize surgical planning and avoid unnecessary procedures.

Recent advances in radiomics offer unprecedented opportunities [10] to decode tumor heterogeneity beyond conventional imaging interpretation [10, 11]. While existing studies primarily focus on intratumoral analysis [12], emerging evidence highlights the complementary prognostic value of peritumoral microenvironment characterization [13]. Tumor‐host interactions within peritumoral regions, manifested as microvascular proliferation, immune cell infiltration, and fibrotic remodeling, provide spatial biomarkers of biological aggressiveness [13]. Notably, radiomic signatures from peritumoral zones have demonstrated diagnostic superiority in discriminating hepatocellular carcinoma from ICC [14] and prediction of ICC grade [15]. While radiomic signatures from peritumoral zones have demonstrated diagnostic value, critical gaps persist in applying this approach to IMCC‐CRLM differentiation. Specifically, comprehensive investigations exploring the impact of varying peritumoral extents remain scarce. Furthermore, existing literature is largely confined to single‐center studies assessing either basic clinical‐imaging features or isolated intralesional radiomics [7, 12], overlooking the microenvironmental dynamics captured by combined intratumoral‐peritumoral radiomic analysis.

In this study, we attempted to develop a novel model that combines multiparametric MRI‐derived radiomic features from the intratumoral and optimal peritumoral regions to achieve a noninvasive differential diagnosis of IMCC and CRLM.

## 2 Methods and Materials

**FIGURE 1:**
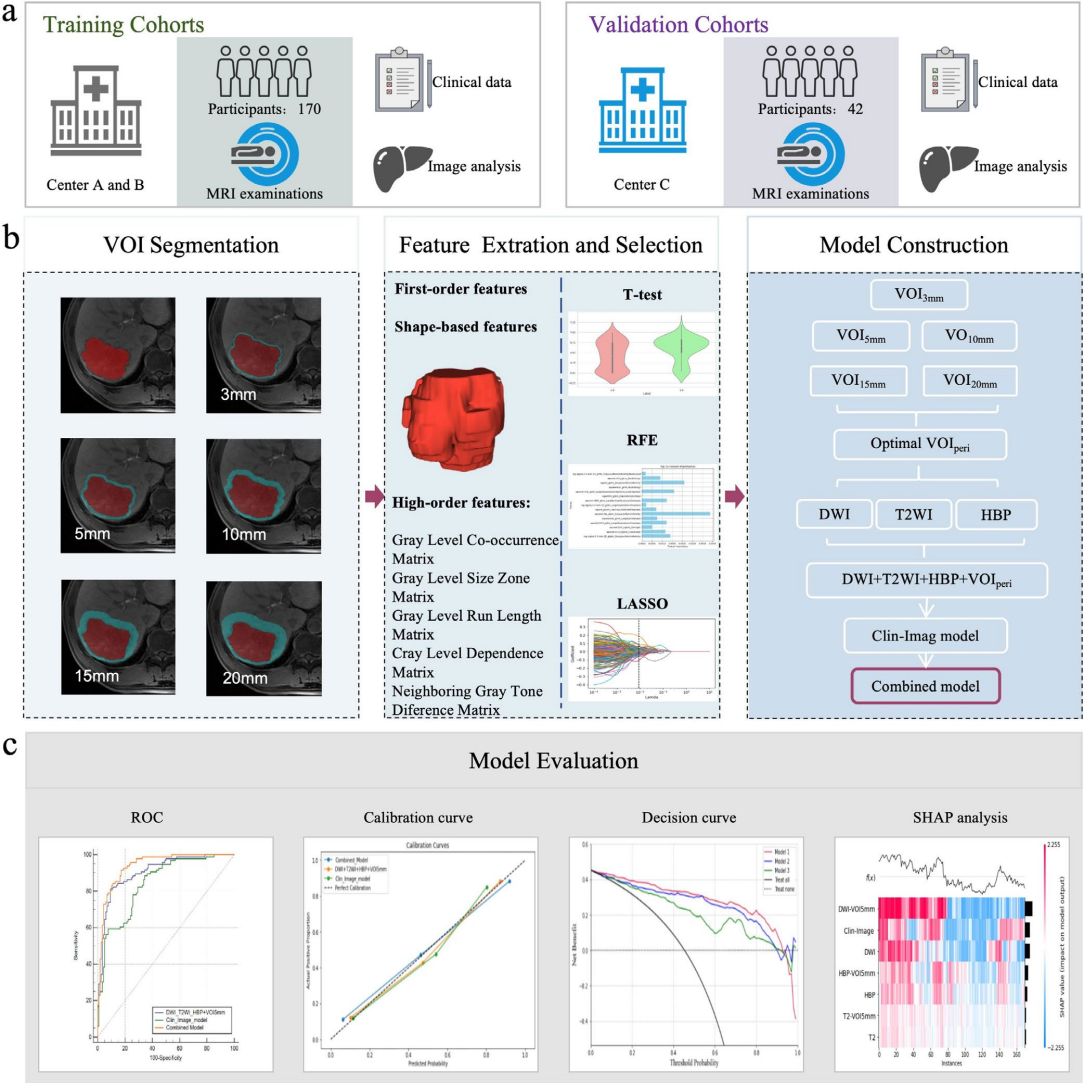
Overview of the study design. (a) The datasets for training were obtained from two distinct medical centers, while the datasets for independent validation were procured from a medical center located in a different area. (b) Radiomics framework of distinguishing intrahepatic mass‐forming cholangiocarcinoma from liver metastasis. (c) Evaluate and compare the diagnostic performance of the clinical‐image model, radiomics model, and combined model. LASSO, least absolute shrinkage and selection operator; RFE, recursive feature elimination; ROC, receiver operating characteristic curve; SHAP, Shapley additive explanations; VOI, volume of interest; VOIperi, VOI peripheral.

This retrospective study was approved by the institutional review board (HDFYLL‐KY‐2024‐021), and the requirement for patient informed consent was waived. The overall research process is shown in Figure 1. This research strictly followed the CLEAR checklist [14].

This study conducted a continuous retrospective review of surgically confirmed IMCC and CRLM patients from three medical centers, spanning from January 2018 to June 2024. Inclusion criteria were as follows: (1) patients had only a single lesion that was histologically confirmed as IMCC or CRLM; (2) patients who underwent liver MRI [T2‐weighted imaging (T2WI), diffusion‐weighted imaging (DWI), and hepatobiliary phase imaging (HBP)] within 1 month prior to surgery; and (3) patients who had not received prior treatment for liver lesions or primary colorectal lesions. Exclusion criteria were as follows: (1) other types of ICC (ICC with periductal infiltration or intraductal growth patterns); (2) missing or out‐of‐interval preoperative MRI data; (3) prior treatment for liver lesions (chemotherapy, radiotherapy, or radiofrequency ablation); and (4) poor MRI image quality due to severe artifacts or noise. Demographic and clinical data were obtained from electronic medical records.

### 2.1 MRI Image Acquisition and Preprocessing

Preoperative abdominal MRI examinations were performed on one of the following MRI scanners: Discovery MR 750 3.0T (GE Healthcare, Milwaukee, WI, USA), uMR 588 1.5T (United Imaging, Shanghai, China), or Signa HDx 3.0 T (GE Healthcare, Milwaukee, WI, USA). The liver examination protocol included axial T2WI, DWI, and HBP (specific scan parameters are shown in Table S1). DWI was performed using respiratory triggering or diaphragm navigation techniques, combined with axial single‐shot spin echo and echo‐planar imaging sequences, as well as diffusion‐weighted gradients applied in three orthogonal directions (*b*‐values of 0 and 800 s/mm [2]). During multiphasic contrast‐enhanced imaging, patients received an intravenous injection of gadobenate dimeglumine (Gd‐BOPTA, Multihance, Bracco Imaging) at a dose of 0.2 mL per kilogram of body weight, administered at a rate of 2 mL per second, followed by a 20 L saline flush. The HBP was acquired 90 min after the start of the Gd‐BOPTA injection.

Before feature extraction, preprocessing was performed on various types of MRI sequences, including DWI, T2WI, and HBP. Specifically, the sitkBSpline interpolation algorithm from the SimpleITK library was used to resample the images, ensuring a consistent resolution of 1 × 1 × 1 mm across all images. The purpose of this step was to eliminate the effects of varying scan resolutions, providing standardized data for subsequent feature extraction.

### 2.2 MRI Image Analysis

Radiological evaluation was conducted on preoperative multiparametric MRI scans by two radiologists: Radiologist 1 (L.Z.), with 10 years of experience in abdominal radiology, and Radiologist 2 (X.L.), with 6 years of experience in the field. Any discrepancies between the two radiologists were resolved by a third senior radiologist (Radiologist 3, L.X.), who had 20 years of experience in abdominal radiology. The final consensus among the three radiologists was reached after their deliberations. Notably, all three radiologists were blinded to the clinical data and pathological results of the patients.

The “target sign” was defined as having a relatively hyperintense peripheral ring or a relatively hyperintense central area. Tumor thrombus refers to the growth or extension of a tumor into adjacent blood vessels. The vascular traversal sign describes the extension of blood vessels into the interior of the tumor. Enlarged lymph nodes were characterized by a short‐axis diameter of 1 cm or more. MRI findings are described based on the following parameters: lesion location (left lobe of the liver, right lobe of the liver, or both), tumor margin (smooth or infiltrative), T2WI tumor boundary (clear or blurry), bile duct dilatation (present or absent), hepatic capsule retraction (present or absent), intrahepatic bile duct stones (present or absent), tumor thrombus (present or absent), vascular traversal sign (present or absent), enlarged lymph nodes (present or absent), DWI signal (target sign and nontarget sign), and T2WI signal (target sign and nontarget sign).

### 2.3 Segmentation of Tumor and Peritumoral Regions

In our study, we utilized ITK‐SNAP (version 4.0.0; http://www.itksnap.org/pmwiki/pmwiki.php) software to delineate the tumor region and peritumoral regions as voxel‐based regions of interest (VOI). Initially, two radiologists with extensive experience in abdominal imaging (Radiologist 1, L.Z., with 10 years of experience in abdominal radiology; Radiologist 2, X.L., with 6 years of experience) meticulously marked the tumor boundaries on the MRI images to define the tumor region. Subsequently, we used the “Edge Expansion” module of 3D‐Slicer software (V5.2.2, https://www.slicer.org) to expand the tumor region (VOItumor) outward by 3 mm (VOI_3mm_), 5 mm (VOI_5mm_), 10 mm (VOI_10mm_), 15 mm (VOI_15mm_), and 20 mm (VOI_20mm_). The tumor portion was then subtracted from the expanded VOI to define the peritumoral region. During this expansion process, the two radiologists carefully removed any parts of the expanded region that fell outside the liver contour to ensure that the peritumoral region contained only liver tissue. Intra‐rater reliability was assessed using the intraclass correlation coefficient (ICC) to evaluate interobserver reproducibility.

### 2.4 Feature Extraction and Selection

Radiomic feature extraction was performed using Pyradiomics [16] software (version 3.0), which extracted 1625 quantitative radiomic features from both intratumoral and peritumoral regions. This included: (1) standard VOIs on DWI, T2WI, and HBP sequences; and (2) 15 extended peritumoral VOIs generated from each of the three baseline sequences. This process involved the application of 15 different filters (Supporting Information), which captured a range of features, including first‐order statistical parameters (*n* = 378), morphological parameters (*n* = 14), gray‐level co‐occurrence matrix (GLCM) parameters (*n* = 441), gray‐level run length matrix (GLRLM) parameters (*n* = 336), gray‐level size zone matrix (GLSZM) parameters (*n* = 336), gray‐level dependence matrix (GLDM) parameters (*n* = 294), and neighboring gray‐tone difference matrix (NGTDM) parameters (*n* = 105).

Initially, we performed variance homogeneity tests and independent samples t‐tests to screen for continuous features that exhibited significant statistical differences (*p* < 0.05) between diseases. Subsequently, we applied recursive feature elimination (RFE) with a fivefold inner cross‐validation loop embedded within the RFE process, iteratively removing features (10% per iteration) that contributed minimally to the model, ultimately retaining the key features that optimized predictive performance. The elimination stopping criterion was defined as the iteration achieving peak mean AUC across the five inner folds. Next, we employed the Least Absolute Shrinkage and Selection Operator (LASSO) regression model, adjusting the regularization parameter α (ranging from 0.0001 to 10 in logarithmic intervals) to automatically select the features crucial for model prediction. Post‐LASSO, variance inflation factors (VIF) confirmed multicollinearity elimination (all VIF < 3).

### 2.5 Model Development

In this study, we employed a logistic regression model as a classifier to differentiate between IMCC and CRLM. To ensure the fairness and accuracy of model evaluation, we utilized a stratified fivefold cross‐validation method, which maintained the proportion of samples from each class consistent with the original dataset in each fold. Additionally, to optimize the regularization strength of the logistic regression model, we constructed a parameter grid in the training set, which included several regularization parameters C (*C* values of 0.01, 0.1, 1, and 10), and systematically searched for the optimal combination using GridSearchCV. Performance for each parameter combination was assessed through internal three‐fold cross‐validation, and the best model configuration was ultimately selected.

The Radiomics model integration protocol was systematically executed through four consecutive phases to optimize multi‐parametric feature utilization. Initially, we performed a comprehensive peritumoral spatial analysis by constructing 15 candidate models (3 sequences × 5 expansion ranges: 3/5/10/15/20 mm) as documented in Table S2. We conducted pairwise DeLong tests comparing ROC curves and then selected the model with the highest predictive accuracy, referred to as the optimal peritumoral model (Table S2). After determining the optimal peritumoral model, we further combined it with the intratumoral models based on DWI, T2WI, and HBP, resulting in three single‐sequence models: DWI + VOI_5mm_, T2WI + VOI_5mm_, and HBP + VOI_5mm_. Moreover, we explored the potential of combining these three single‐sequence models to conduct the Radiomics model (DWI + T2WI + HBP + VOI_5mm_). Our Radiomics integration framework uniformly employed stacking‐based probabilistic ensemble strategies across both intra‐ and intersequence modeling phases.

For the Clin‐Imag model, we used univariate logistic regression analysis to evaluate differences in clinical and imaging characteristics between IMCC and CRLM patients in the training cohort. Variables with a *p* value less than 0.05 were included in the multivariate logistic regression analysis, and the statistically significant results were incorporated into the development of the clinical‐imaging model.

Finally, we integrated the radiomics model with a Clin‐Imag model to construct the final combined model.

### 2.6 Statistical Analysis

Continuous variables and categorical variables were reported as median with standard deviation and frequency with percentage, respectively. Fisher's exact test or Pearson's chi‐square test, along with the Kruskal–Wallis test, were used to compare patient characteristics in both the training and external test datasets. Univariate and multivariate logistic regression analyses were conducted to evaluate differences in clinical and imaging features between IMCC and CRLM. The model's predictive performance in distinguishing between IMCC and CRLM was assessed using the receiver operating characteristic (ROC) curve, along with the area under the curve (AUC) and its 95% confidence interval (CI). The diagnostic performance among different models was compared using the DeLong test, two‐sided, with statistical significance set at *p* < 0.05. Model calibration was evaluated using the Hosmer–Lemeshow test, and decision curve analysis was performed to measure the clinical utility of the predictive models. Additionally, various performance metrics, including accuracy, sensitivity, specificity, positive predictive value (PPV), and negative predictive value (NPV), were calculated. To analyze the contribution of various features to the model's predictions, Shapley additive explanations (SHAP) analysis was conducted.

## 3 Results

### 3.1 Baseline Characteristics and Clin‐Imag Model Construction

**FIGURE 2:**
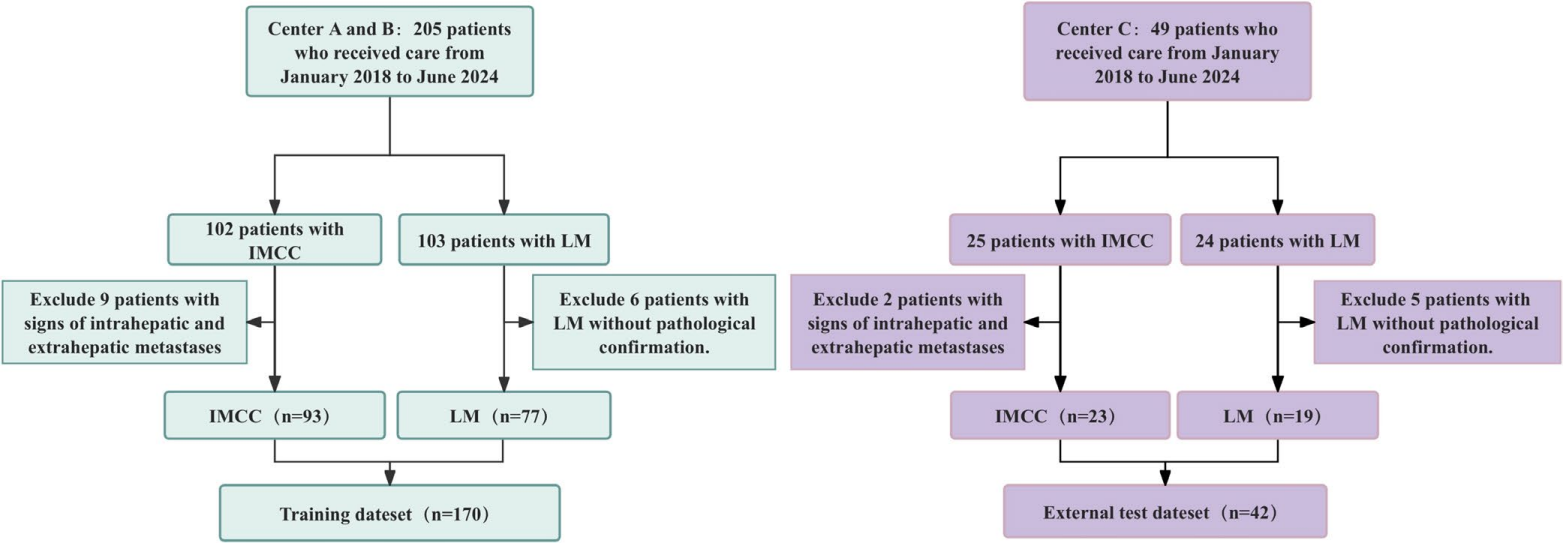
Flowchart of the patient inclusion and exclusion process. IMCC, intrahepatic mass‐forming cholangiocarcinoma; LM, liver metastases.

A total of 170 patients (93 with IMCC and 77 with CRLM) were included in the training cohort, and 42 patients (23 with IMCC and 19 with CRLM) were included in the external validation cohort (Figure 2). A summary of comparisons of demographic, clinical, and radiological characteristics is presented in Table 1. As shown in Table 2, univariate analysis revealed significant differences between the IMCC and CRLM groups in the training cohort for lesion location, tumor margin, T2WI tumor boundary, bile duct dilation, hepatic capsule retraction, vascular involvement, and vascular traversal sign (*p* < 0.001, *p* < 0.001, *p* < 0.001, *p* < 0.001, *p* < 0.001, *p* < 0.001, *p* = 0.001). Multivariate logistic regression analysis identified lesion location, tumor margin, T2WI tumor boundary, and bile duct dilation as independent factors for distinguishing IMCC from CRLM (*p* = 0.001, *p* = 0.010, *p* = 0.022, *p* = 0.002).

**TABLE 1:** Comparative analysis of clinical, laboratory, and imaging features between the training and external validation cohorts.

Characteristic	Training cohorts			External validation cohorts			*p*
	IMCC (*n* = 93)	CRLM (*n* = 77)	*p*	IMCC (*n* = 23)	CRLM (*n* = 19)	*p*	
Age (year)	61.280 ± 34.870	61.060 ± 36.120	0.967	61.440 ± 10.570	61.500 ± 10.290	0.985	0.953
Sex			1			1	0.919
Male	40 (43.000)	31 (40.260)		9 (39.130)	6 (31.579)		
Female	53 (56.989)	46 (59.740)		14 (60.870)	13 (68.421)		
Lesion Location			1			1	1
Left Lobe	30 (32.258)	0 (0.000)		5 (21.739)	1 (5.263)		
Right Lobe	60 (64.516)	35 (45.455)		16 (69.565)	6 (31.579)		
Both	3 (3.226)	42 (54.545)		2 (8.696)	12 (63.158)		
Tumor margin			1			1	0.834
Smooth	54 (58.065)	70 (90.909)		15 (65.217)	15 (78.947)		
Infiltrative	39 (41.935)	7 (9.091)		8 (34.783)	4 (21.053)		
T2WI tumor boundary			1			1	0.506
Clear	35 (37.634)	72 (93.506)		14 (60.870)	16 (84.211)		
Blurry	58 (62.366)	5 (6.494)		9 (39.130)	3 (15.789)		
Bile duct dilatation	89 (95.699)	27 (35.065)	1	20 (86.957)	9 (47.368)	1	0.386
Hepatic capsule retraction	90 (96.774)	48 (62.338)	1	20 (86.957)	10 (52.632)	1	0.310
Intrahepatic bile duct stones	93 (100.000)	73 (94.805)	1	23 (100.000)	18 (94.737)	1	1
Vascular involvement	90 (96.774)	33 (42.857)	1	23 (100.000)	18 (94.737)	1	1
Vascular traversal sign	91 (97.849)	59 (76.623)	1	22 (95.652)	15 (78.947)	1	1
Suspicious lymph nodes	74 (79.570)	59 (76.623)	1	19 (82.609)	13 (68.421)	1	1
T2WI signal pattern			1			1	1
Target sign	60 (64.516)	45 (58.442)		11 (47.826)	11 (57.895)		
No‐target sign	33 (35.484)	32 (41.558)		12 (52.174)	8 (42.105)		
DWI signal pattern			1			1	0.516
Target sign	47 (50.538)	38 (49.351)		10 (43.478)	10 (52.632)		
No‐target sign	46 (49.462)	39 (50.649)		13 (56.522)	9 (47.368)		
HBP signal pattern			1			1	0.266
Target sign	77 (82.796)	67 (87.013)		10 (43.478)	11 (57.895)		
No‐target sign	16 (17.204)	10 (12.987)		13 (56.522)	8 (42.105)		

Abbreviations: CRLM, colorectal cancer liver metastases; DWI, diffusion‐weighted imaging; HBP, hepatobiliary phase; IMCC, intrahepatic mass‐forming cholangiocarcinoma; T2WI, T2‐weighted imaging.

**TABLE 2:** Univariate and multivariable logistic regression analysis for distinguishing intrahepatic mass‐forming cholangiocarcinoma from colorectal cancer liver metastases.

Variable	Univariate analysis			Multivariate analysis		
	Odds ratio	(95% CI)	*p*	Odds ratio	(95% CI)	*p*
Age (year)	1.001	0.975–1.027	0.966			
Sex	1.166	0.672–2.025	0.585			
Lesion location	19.780	8.321–47.017	**< 0.001** *	107.220	7.558–1521.138	**0.001** *
Tumor margin	0.190	0.092–0.394	**< 0.001** *	0.003	0.000–0.251	**0.010** *
T2WI tumor boundary	0.067	0.030–0.150	**< 0.001** *	0.021	0.001–0.573	**0.022** *
Bile duct dilatation	25.952	10.886–61.871	**< 0.001** *	29.400	3.45–250.60	**0.002** *
Hepatic capsule retraction	12.016	4.797–30.077	**< 0.001** *	1.895	0.134–26.726	0.636
Vascular involvement	141.74	19.008–1057.021	**< 0.001** *	24.903	0.520–1193.131	0.103
Vascular traversal Sign	11.198	3.236–38.751	**0.001** *	3.416	0.109–106.863	0.484
Suspicious lymph nodes	1.348	0.704–2.580	0.368			
T2WI signal pattern	1.070	0.909–1.261	0.416			
DWI signal pattern	0.894	0.667–1.199	0.455			
HBP signal pattern	0.899	0.622–1.300	0.571			

*Note:* *p < 0.05; boldface type indicates *p* < 0.05.

Abbreviations: CI, confidence interval; DWI, diffusion‐weighted imaging; HBP, hepatobiliary phase; T2WI, T2‐weighted imaging.

The Clin‐Imag model achieved an AUC of 0.858 (95% CI: 0.831–0.880) and an accuracy of 0.750 in the training cohort. In the external validation cohort, the AUC was 0.842 (95% CI: 0.786–0.889), with an accuracy of 0.745 (Table 3).

**TABLE 3:** Discrimination performance comparison of the prediction models for distinguishing intrahepatic mass‐forming cholangiocarcinoma from colorectal cancer liver metastases.

Models		Feature count	AUC	Accuracy	Sensitivity	Specificity	PPV	NPV
DWI	Training cohorts	21	0.904 (95% CI: 0.882, 0.923)	0.810	0.760	0.851	0.809	0.811
	External test cohorts	21	0.805 (95% CI 0.805, 0.903)	0.736	0.667	0.793	0.727	0.742
T2WI	Training cohorts	16	0.833 (95% CI: 0.805, 0.858)	0.772	0.659	0.866	0.803	0.754
	External test cohorts	16	0.816 (95% CI: 0.757, 0.866)	0.769	0.677	0.845	0.783	0.760
HBP	Training cohorts	17	0.832 (95% CI: 0.802, 0.857)	0.730	0.672	0.778	0.803	0.741
	External test cohorts	17	0.805 (95% CI: 0.745, 0.856)	0.698	0.625	0.759	0.682	0.710
DWI + VOI_5mm_	Training cohorts	35	0.945 (95% CI: 0.931, 0.960)	0.872	0.839	0.901	0.875	0.871
	External test cohorts	35	0.885 (95% CI: 0.834, 0.924)	0.783	0.729	0.828	0.778	0.787
T2WI + VOI_5mm_	Training cohorts	17	0.834 (95% CI: 0.805, 0.860)	0.788	0.688	0.871	0.815	0.771
	External test cohorts	17	0.817 (95% CI: 0.758, 0.867)	0.774	0.667	0.862	0.800	0.758
HBP + VOI_5mm_	Training cohorts	17	0.866 (95% CI: 0.842, 0.890)	0.787	0.740	0.825	0.778	0.793
	External test cohorts	17	0.838 (95% CI: 0.781, 0.885)	0.769	0.729	0.802	0.753	0.782
DWI + T2WI + HBP	Training cohorts	25	0.835 (95% CI: 0.807, 0.860)	0.769	0.669	0.851	0.788	0.757
	External test cohorts	25	0.830 (95% CI: 0.773, 0.882)	0.736	0.646	0.810	0.738	0.734
DWI + T2WI + HBP + VOI_5mm_	Training cohorts	3	0.947 (95% CI: 0.932, 0.961)	0.860	0.841	0.875	0.848	0.869
	External test cohorts	3	0.908 (95% CI:0.861, 0.943)	0.832	0.823	0.862	0.832	0.855
Clin‐Imag model	Training cohorts	4	0.858 (95% CI: 0.831, 0.880)	0.750	0.799	0.709	0.694	0.810
	External test cohorts	4	0.842 (95% CI 0.786, 0.889)	0.745	0.792	0.707	0.691	0.804
Combined model	Training cohorts	7	0.978 (95% CI: 0.971, 0.985)	0.917	0.924	0.912	0.896	0.936
	External test cohorts	7	0.940 (95% CI:0.899, 0.968)	0.868	0.823	0.897	0.868	0.860

Abbreviations: AUC, area under the receiver operating characteristic curve; CI, confidence intervals; NPV, negative predictive value; PPV, positive predictive value; VOI, volumes of interest.

### 3.2 Radiomics Model

As shown in Table S2 and Figure S1, a total of 18 models based on different peritumoral ranges for DWI, T2WI, and HBP were evaluated in the training cohort. The DeLong test was used to compare the predictive performance of different models, and the results demonstrated that the model based on the 5 mm tumor‐periphery range in DWI achieved the best predictive performance, with an AUC of 0.943 (95% CI: 0.926–0.957). Therefore, VOI_5mm_ was chosen as the optimal peritumoral range.

Furthermore, as shown in Table 3, among the three single‐sequence models based on DWI + VOI_5mm_, T2WI + VOI_5mm_, and HBP + VOI_5mm_, the DWI + VOI_5mm_ model exhibited the highest predictive efficiency, with an AUC of 0.945 (95% CI: 0.931–0.960) in the training cohort and an AUC of 0.885 (95% CI: 0.834–0.924) in the external validation cohort.

The intratumoral multi‐sequence model (DWI + T2WI + HBP) achieved baseline AUCs of 0.835 (95% CI: 0.807–0.860) in the training cohort and 0.830 (95% CI: 0.773–0.882) in external validation. After integrating 5‐mm peritumoral Radiomics features (DWI + T2WI + HBP + VOI5 mm), the final Radiomics model demonstrated significantly enhanced performance, with AUC improvements to 0.947 (95% CI: 0.932–0.961) and 0.908 (95% CI: 0.861–0.943) in training and validation cohorts, respectively (Table 3). Critical diagnostic parameters also improved substantially: sensitivity increased by 17.2% (training) and 17.7% (validation), specificity rose 2.4% (training) and 5.2% (validation), and accuracy improved by 9.1% (training) and 9.6% (validation) in both cohorts compared to the intratumoral‐only model (DWI + T2WI + HBP).

### 3.3 Performance Evaluation of Prediction Models

**FIGURE 3:**
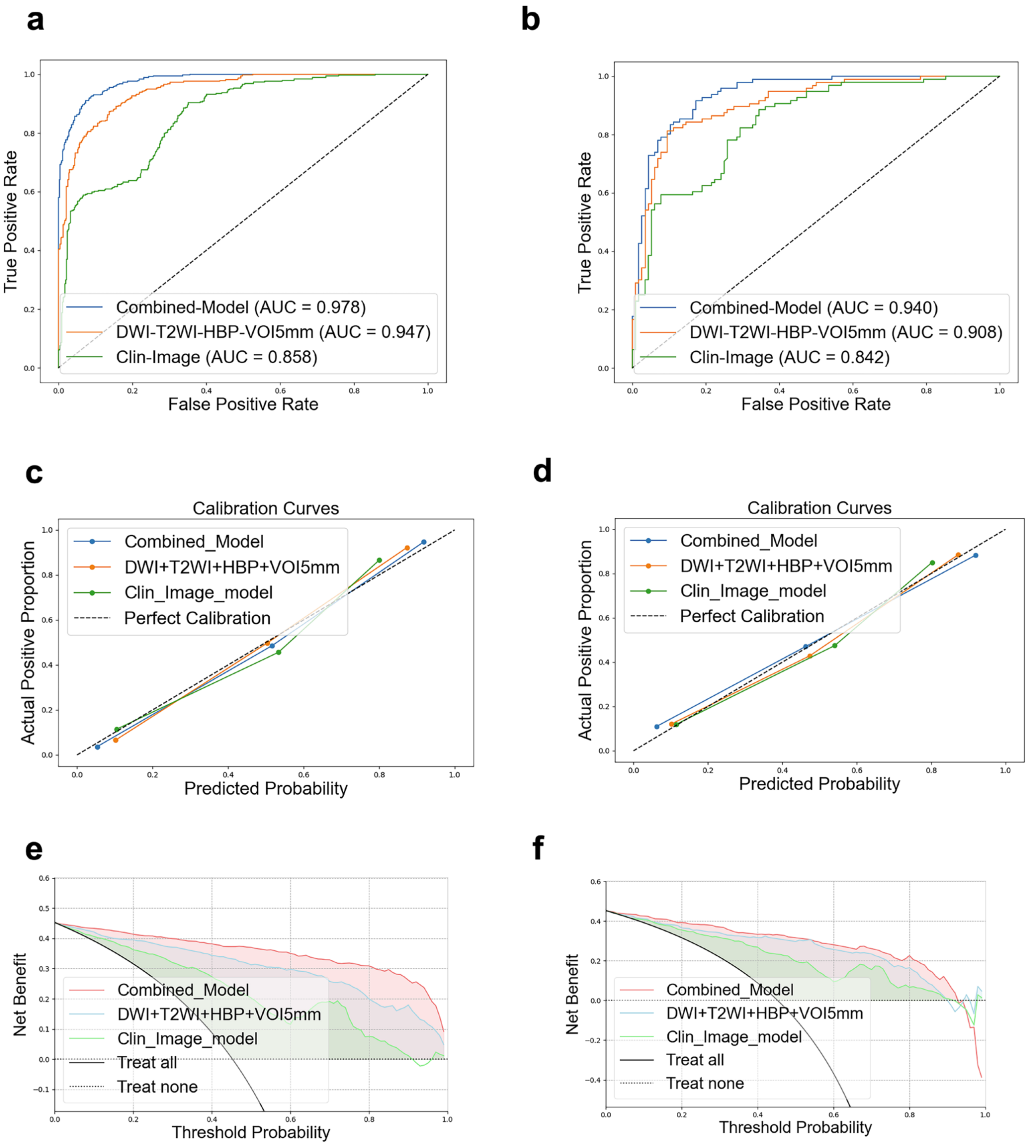
The receiver operating characteristic curves (a, b), calibration curves (c, d), and decision curve analysis (e, f) of the different models in the training cohort and external validation cohort. AUC, area under the curve.

The Combined model demonstrated the highest discriminative performance among all models (Figure 3). In the training cohort, the AUC was 0.978 (95% CI: 0.971–0.985) with an accuracy of 0.917. In the external validation cohort, the AUC was 0.940 (95% CI: 0.899–0.968) with an accuracy of 0.868.

**FIGURE 4:**
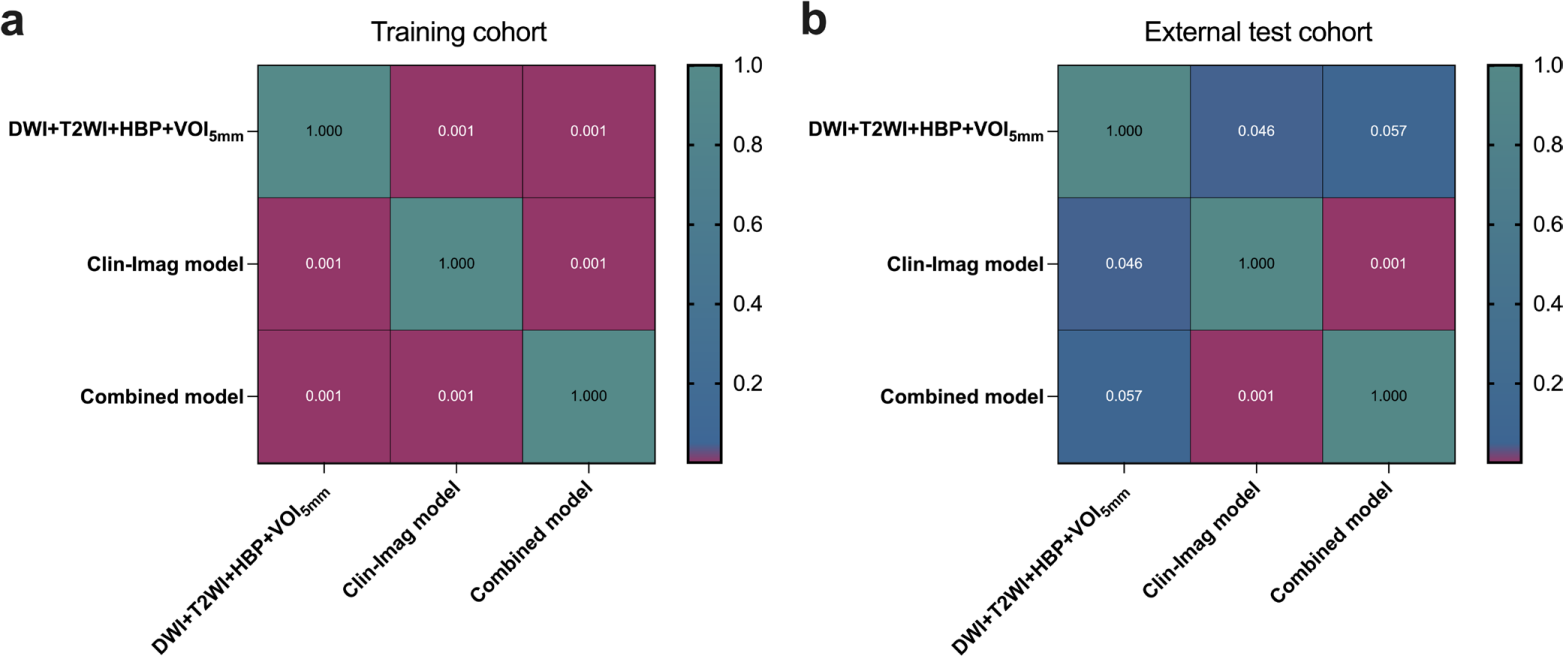
Heatmaps of Delong test in the training cohort (a) and external validation cohort (b).

DeLong test results (Figure 4) showed that in the external validation cohort, the combined model outperformed the Clin‐Imag model in terms of discriminative performance (AUC: 0.940 vs. 0.842; *p* = 0.001). Although no statistically significant difference was found between the combined model and the radiomics model (AUC: 0.940 vs. 0.908; *p* = 0.057), the combined model achieved a higher accuracy of 0.868 compared to 0.832 for the radiomics model. Furthermore, the radiomics model showed superior diagnostic performance compared to the Clin‐Imag model (AUC: 0.908 vs. 0.842; *p* = 0.046).

**FIGURE 5:**
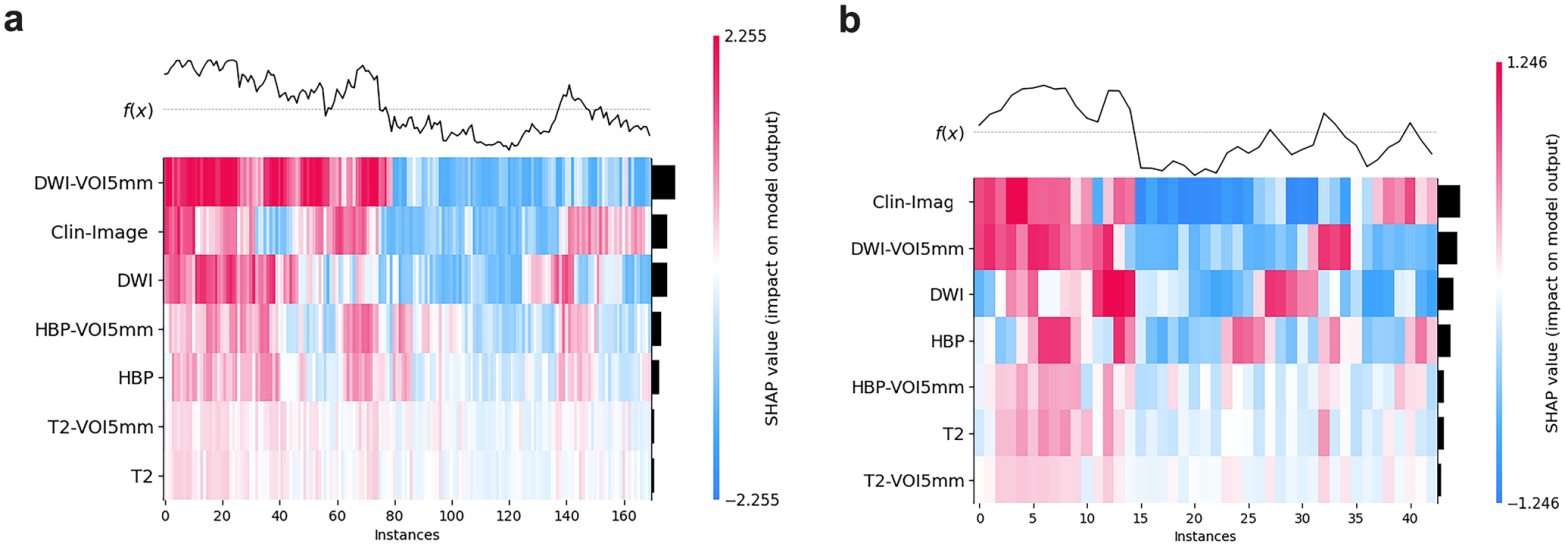
SHapley additive explanations (SHAP) summary plot illustrating the impact of various medical imaging and clinical features on the output of a machine learning model. Each feature's contribution is represented by SHAP values, with red indicating positive impact and blue indicating negative impact on the model prediction. High‐impact features such as DWI‐VOI_5mm_ and Clin‐Image show significant variation across instances, suggesting a strong influence on the model's prediction.

**FIGURE 6:**
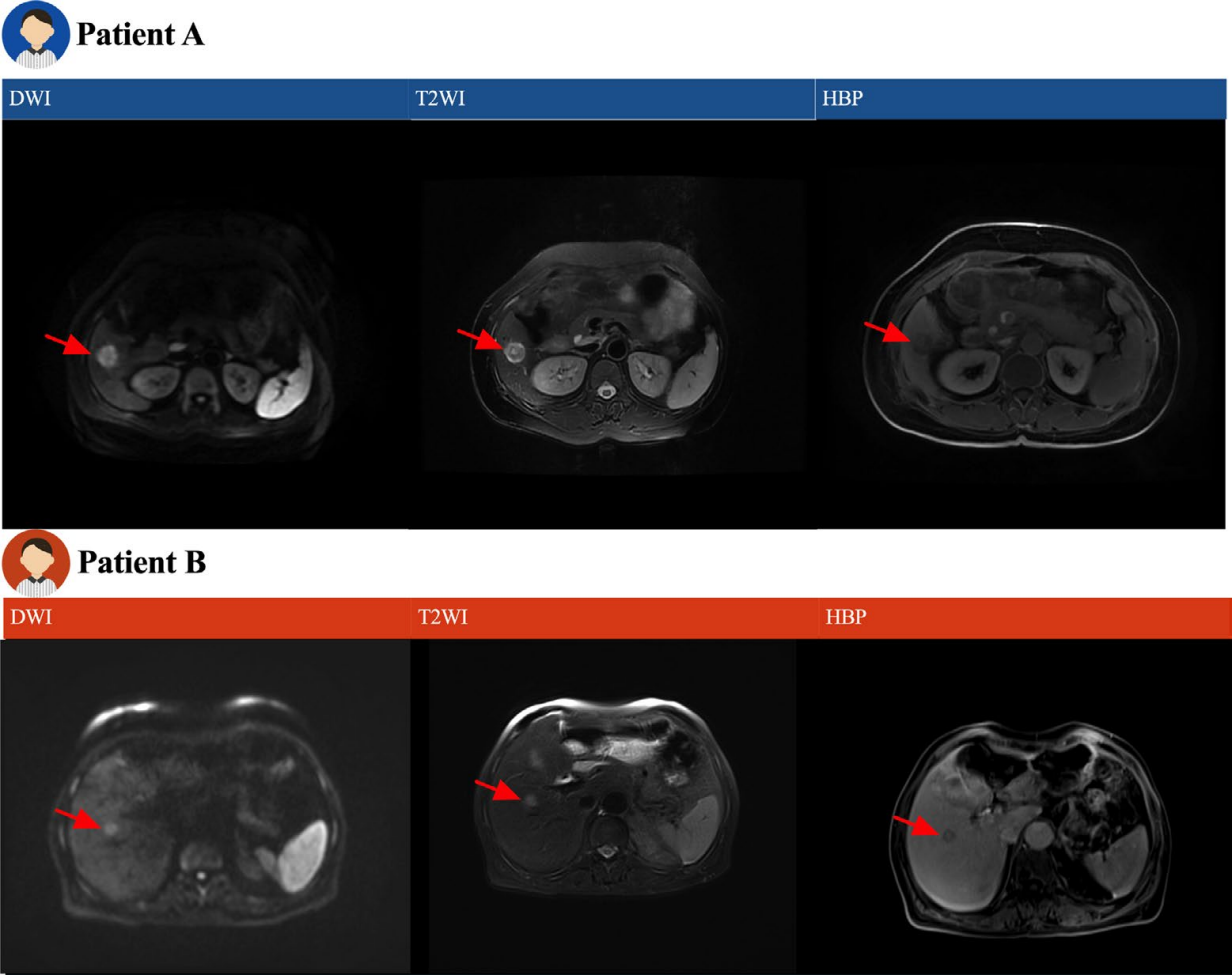
Representative cases of intrahepatic mass‐forming cholangiocarcinoma (IMCC) and colorectal liver metastasis (CRLM). Patient A: A 55‐year‐old male diagnosed with IMCC. Patient B: A 48‐year‐old male diagnosed with CRLM. DWI, diffusion‐weighted imaging; HBP, hepatobiliary phase; T2WI, T2‐weighted imaging.

Additionally, Shapley additive explanations (SHAP) analysis of the combined model (Figure 5) revealed that both the Clin‐Imag model and DWI‐VOI_5mm_ exhibited significant positive influence in both the training and external validation cohorts. A representative case is illustrated in Figure 6.

## 4 Discussion

In this study, we developed a comprehensive model for the preoperative differentiation of IMCC and CRLM based on the optimal peritumoral range (5 mm) and multi‐parametric MRI radiomic features. This model demonstrated strong discriminatory ability and high reliability, and it was validated using an independent external cohort. SHAP analysis revealed that key features, including lesion location, tumor margin, T2WI tumor boundary, bile duct dilation, and radiomic features derived from the DWI with a 5 mm peritumoral range, exhibited significant positive influence and were crucial factors in the model's decision‐making process.

Several studies have shown that MRI‐based predictive models can be used to differentiate IMCC from CRLM [7, 17]. However, the delineated VOIs in these models are typically based on the lesion itself, which may overlook the additional information provided by the tumor surrounding region regarding tumor invasiveness and behavior [13]. In contrast, we innovatively extracted radiomic features from both the tumor and its surrounding regions. We found that the optimal VOI scale was 5 mm, which includes the 5 mm peripheral area around the tumor margin. This suggests that the transitional zone within the 5 mm tumor margin contains microenvironmental information relevant for differentiating IMCC from CRLM [18], such as inflammatory cells involved in the immune response [19], including macrophages and T lymphocytes. SHAP analysis indicated that these features are crucial for the predictive capability of the Radiomics model.

Unlike previous studies on predicting microvascular invasion in IMCC [20], which identified the optimal tumor‐periphery range as 10 mm, our finding of a 5 mm margin may be due to differences in the focus of the single lesion and varying depths of tumor invasion.

Consistent with previous studies on different tumors, integrating the 5 mm peripheral region with tumor features significantly enhances the prediction of tumor characteristics. This integrated model provides more accurate predictions for early and late‐stage tumor recurrence [21], as well as tumor grading and microvascular invasion [22]. Furthermore, a study exploring the relationship between peripheral liver parenchyma MRI signals in CRLM using gadolinium‐ethoxybenzyl‐diethylenetriamine pentaacetic acid contrast enhancement and pathological vascular invasion and long‐term prognosis [23] demonstrated that changes in peripheral MRI signal intensity could predict long‐term prognosis after curative surgery without neoadjuvant chemotherapy. Thus, integrating radiomic features from both the intratumoral and peritumoral radiomics regions into clinical decision‐making supports more accurate and noninvasive predictions, potentially improving clinical outcomes for patients [22].

The results of the multivariate logistic regression analysis indicated that lesion location, tumor margin, T2WI tumor boundary, and bile duct dilation are independent factors for differentiating IMCC from CRLM. This finding is consistent with previous studies [23, 24, 25]. In their research [24], IMCC typically exhibits irregular or lobulated margins, which are likely associated with the tumor's aggressive growth pattern, leading to an irregular contour at the tumor boundary. In contrast, CRLM tends to demonstrate clearer and more regular boundaries, possibly due to the way colorectal cancer cells metastasize within the liver, often forming more regular nodular structures. Regarding T2WI imaging characteristics [24], IMCC is often more heterogeneous internally, potentially containing areas of necrosis, fibrotic tissue, or cells with varying densities. This heterogeneity results in a highly heterogeneous signal on T2WI, making the tumor boundaries relatively well‐defined. In contrast, CRLM may show more homogeneous signal characteristics with less obvious heterogeneity, leading to a relatively blurred boundary on T2WI. These independent factors could provide new perspectives for the radiological diagnosis of liver lesions. Our study also demonstrated that among the three MRI sequences—DWI, T2WI, and HBP—DWI is the most effective sequence for distinguishing IMCC from CRLM, with its high sensitivity and accuracy being well‐supported. Similar to earlier findings, DWI has been highlighted for its strong predictive value in differentiating IMCC from other diseases, such as atypical hepatic abscesses [26, 27]. This underscores DWI's importance as a valuable tool for differentiating various types of liver lesions.

This study innovatively established a multiparametric MRI‐based Radiomics model through multimodal ensemble learning integrating DWI, T2WI, and HBP sequences. The model demonstrated robust discriminatory performance with AUCs of 0.947 (95% CI: 0.932–0.961) and 0.908 (95% CI: 0.861–0.943) in training and external validation cohorts, respectively. By probabilistically aggregating complementary information from three distinct MRI sequences: DWI for cellular density characterization, T2WI for fluid‐sensitive tissue discrimination, and HBP for hepatobiliary functional assessment, the ensemble framework effectively captures multidimensional tumor heterogeneity. This outperformed both single‐sequence models and conventional Clin‐Imag approaches, aligning with Xu et al. [12, 15] who emphasized multimodal integration for compensating class‐imbalanced features in hepatic lesion differentiation. Such multiparametric interpretability supports clinical translation by mapping model decisions to recognizable biological concepts. While further validation is warranted, this multimodal radiomics paradigm establishes a framework for precision hepatic oncology, potentially reducing unnecessary invasive procedures through reliable non‐invasive characterization of lesions.

## 5 Limitations

First, this retrospective study may have potential selection bias, and future research should focus on expanding the sample size. Second, although preoperative imaging of IMCC and CRLM was assessed, long‐term follow‐up is necessary to observe the relationships between imaging features, Radiomics features, clinical outcomes, and survival. Third, our study utilized manual segmentation with fixed peritumoral expansions, which highlights the broader challenge of protocol standardization in radiomics. The absence of consensus guidelines for peritumoral VOI definition limits cross‐study comparability. We advocate for international consensus to establish unified protocols enabling multi‐center validation and clinical translation. While external validation strengthens our findings, the cohort size limits statistical power for subgroup analyses. Future multicenter studies with larger cohorts are needed. Finally, despite standardized preprocessing, vendor‐specific differences in field strength, coil configurations, and reconstruction algorithms may persist. Advanced harmonization techniques are needed for vendor‐agnostic models.

## 6 Conclusion

We have developed and validated a combined model that integrates clinical, imaging features, and both intratumoral and optimal peritumoral range radiomics features. This model was shown to be more effective in distinguishing IMCC from CRLM in an external validation cohort compared to independent radiomics or Clin‐Imag models. Radiomics features from the tumor‐peripheral region, especially the 5 mm region, play a critical role in enhancing the predictive ability for distinguishing IMCC and CRLM.

## Author Contributions

Conceptualization, L.Z. and X.L.; image segmentation, S.D. and Y.L.; data analysis, L.X. and Z.S.; data curation, J.W.; writing – original draft, L.Z.; supervision, X.Y. and C.L.

## Ethics Statement

The study was conducted in accordance with the Declaration of Helsinki. This retrospective study was approved by the Ethics Committee of Affiliated Hospital of Hebei University (HDFYLL‐KY‐2024‐021), and the requirement for written informed consent was waived.

## Conflicts of Interest

The authors declare no conflicts of interest.

## Supporting information


Data S1.

## Data Availability

The data that support the findings of this study are available on request from the corresponding author. The data are not publicly available due to privacy or ethical restrictions.
