# Supplementary material for: Multiparametric MRI‐Based Intratumoral and Peritumoral Radiomics for Distinguishing Solitary Intrahepatic Mass‐Forming Cholangiocarcinoma From Colorectal Liver Metastases
Source: Cancer Med. 2025 Aug 5;14(15):e71120. doi: 10.1002/cam4.71120 (PMC12322924; doi:10.1002/cam4.71120)
Supplement: Supplementary file 1 — Data S1. [file CAM4-14-e71120-s001.docx]

**MRI Image Filters**

# Additive Gaussiannoise Filter

Alter an image with additive Gaussian white noise.Additive Gaussian white noise can be modeled as:

$$I=I_{0}+N$$

Where $I$ is the observed image, $I_{0}$ is the noise-free image and $N$is a normally distributed random variable of mean $\sigma^{2}$ and variance :

$$N～N(\mu,\sigma^{2})$$

The noise is independent of the pixel intensities.

# Bilateral Filter

This filter uses bilateral filtering to blur an image using both domain and range "neighborhoods". Pixels that are close to a pixel in the image domain and similar to a pixel in the image range are used to calculate the filtered value. Two gaussian kernels (one in the image domain and one in the image range) are used to smooth the image. The result is an image that is smoothed in homogeneous regions yet has edges preserved. The result is similar to anisotropic diffusion but the implementation in non-iterative. Another benefit to bilateral filtering is that any distance metric can be used for kernel smoothing the image range. Hence, color images can be smoothed as vector images, using the CIE distances between intensity values as the similarity metric (the Gaussian kernel for the image domain is evaluated using CIE distances). A separate version of this filter will be designed for color and vector images.Bilateral filtering is capable of reducing the noise in an image by an order of magnitude while maintaining edges.

# Binomial Blur Image Filter

Performs a separable blur on each dimension of an image.The binomial blur consists of a nearest neighbor average along each image dimension. The net result after n-iterations approaches convultion with a gaussian.

# Box Mean Filter

Implements a fast rectangular mean filter using the accumulator approach.

# Box Sigma Image Filter

Implements a fast rectangular sigma filter using the accumulator approach.

# Curvature Flow Filter

Denoise an image using curvature driven flow[.CurvatureFlowImageFilter](https://itk.org/SimpleITKDoxygen/html/classitk_1_1simple_1_1CurvatureFlowImageFilter.html) implements a curvature driven image denoising algorithm. Iso-brightness contours in the grayscale input image are viewed as a level set. The level set is then evolved using a curvature-based speed function:

$$I_{k}=_{k} \backslash\nabla I\backslash$$

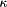
where is the curvature.

The advantage of this approach is that sharp boundaries are preserved with smoothing occurring only within a region. However, it should be noted that continuous application of this scheme will result in the eventual removal of all information as each contour shrinks to zero and disappear.

Note that unlike level set segmentation algorithms, the image to be denoised is already the level set and can be set directly as the input using the SetInput() method.

This filter has two parameters: the number of update iterations to be performed and the timestep between each update.

The timestep should be "small enough" to ensure numerical stability. Stability is guarantee when the timestep meets the CFL (Courant-Friedrichs-Levy) condition. Broadly speaking, this condition ensures that each contour does not move more than one grid position at each timestep. In the literature, the timestep is typically user specified and have to manually tuned to the application.

This filter make use of the multi-threaded finite difference solver hierarchy. Updates are computed using a [Curvature Flow Function](https://www.itk.org/Doxygen/html/classitk_1_1CurvatureFlowFunction.html) object. A zero flux Neumann boundary condition when computing derivatives near the data boundary.

This filter may be streamed. To support streaming this filter produces a padded output which takes into account edge effects. The size of the padding is m_NumberOfIterations on each edge. Users of this filter should only make use of the center valid central region.

# Discrete Gaussian Filter

Blurs an image by separable convolution with discrete gaussian kernels. This filter performs Gaussian blurring by separable convolution of an image and a discrete Gaussian operator (kernel).The Gaussian operator used here was described by Tony Lindeberg (Discrete Scale-Space Theory and the Scale-Space Primal Sketch. Dissertation. Royal Institute of Technology, Stockholm, Sweden. May 1991.) The Gaussian kernel used here was designed so that smoothing and derivative operations commute after discretization.

# Laplacian Sharpening Filter

This filter sharpens an image using a Laplacian. LaplacianSharpening highlights regions of rapid intensity change and therefore highlights or enhances the edges. The result is an image that appears more in focus.

# Mean Filter

Applies an averaging filter to an image.Computes an image where a given pixel is the mean value of the the pixels in a neighborhood about the corresponding input pixel.A mean filter is one of the family of linear filters.

# Median Filter

Applies a median filter to an image.Computes an image where a given pixel is the median value of the the pixels in a neighborhood about the corresponding input pixel.A median filter is one of the family of nonlinear filters. It is used to smooth an image without being biased by outliers or shot noise.

# Normalize Filter

Normalize an image by setting its mean to zero and variance to one. NormalizeImageFilter shifts and scales an image so that the pixels in the image have a zero mean and unit variance. This filter uses StatisticsImageFilter to compute the mean and variance of the input and then applies ShiftScaleImageFilter to shift and scale the pixels.NB: since this filter normalizes the data to lie within -1 to 1, integral types will produce an image that DOES NOT HAVE a unit variance.

# Recursive Gaussian Filter

Base class for computing IIR convolution with an approximation of a Gaussian kernel.

$$\frac{1}{\sigma\sqrt{2\pi}}\exp(-\frac{x^{2}}{2\sigma^{2}})$$

RecursiveGaussianImageFilter is the base class for recursive filters that approximate convolution with the Gaussian kernel. This class implements the recursive filtering method proposed by R.Deriche in IEEE-PAMI Vol.12, No.1, January 1990, pp 78-87, "Fast Algorithms for Low-Level Vision"

# Shot Noise Filter

Alter an image with shot noise.The shot noise follows a Poisson distribution:

$$I=N(I_{0})$$

where $N(I_{0})$ is a Poisson-distributed random variable of mean $I_{0}$. The noise is thus dependent on the pixel intensities in the image.

The intensities in the image can be scaled by a user provided value to map pixel values to the actual number of particles. The scaling can be seen as the inverse of the gain used during the acquisition. The noisy signal is then scaled back to its input intensity range:

where $\delta$ is the scale factor.$I=\frac{N(I_{0}\times\delta)}{\delta}$

The Poisson-distributed variable $\lambda$ is computed by using the algorithm:

$$\begin{matrix} k\leftarrow0 \\ p\leftarrow1 \\ \mathrm{repeat}\left\{ \begin{aligned} k=k+1 \\ p=p*U() \end{aligned} \right. \\ untilp>e^{\lambda},reture(k) \end{matrix}$$

where $U()$ provides a uniformly distributed random variable in the interval [0,1] .

This algorithm is very inefficient for large values of $\lambda$ , though. Fortunately, the Poisson distribution can be accurately approximated by a Gaussian distribution of mean and variance $\lambda$ when$\lambda$ is large enough. In this implementation, this value is considered to be 50. This leads to the faster algorithm:

$$\lambda+\sqrt{\lambda}\times N()$$

where $N()$ is a normally distributed random variable of mean 0 and variance 1.

# Smoothing Recursive Gaussian Filter

Computes the smoothing of an image by convolution with the Gaussian kernels implemented as IIR filters.This filter is implemented using the recursive gaussian filters. For multi-component images, the filter works on each component independently.For this filter to be able to run in-place the input and output image types need to be the same and/or the same type as the RealImageType.

# Speckle Noise Filter

Alter an image with speckle (multiplicative) noise.The speckle noise follows a gamma distribution of mean 1 and standard deviation provided by the user. The noise is proportional to the pixel intensity.

It can be modeled as:

$$I=I_{0}*G$$

where $G$is a is a gamma distributed random variable of mean 1 and variance proportional to the noise level:

$$G\sim\Gamma(\frac{1}{\sigma^{2}},\sigma^{2})$$

# WaveletFilter

A wavelet is a wave-like oscillation with an amplitude that begins at zero, increases, and then decreases back to zero. It can typically be visualized as a "brief oscillation" like one recorded by a seismograph or heart monitor. Generally, wavelets are intentionally crafted to have specific properties that make them useful for signal processing. Using a "reverse, shift, multiply and integrate" technique called convolution, wavelets can be combined with known portions of a damaged signal to extract information from the unknown portions.

For example, a wavelet could be created to have a frequency of Middle C and a short duration of roughly a 32nd note. If this wavelet were to be convolved with a signal created from the recording of a song, then the resulting signal would be useful for determining when the Middle C note was being played in the song. Mathematically, the wavelet will correlate with the signal if the unknown signal contains information of similar frequency. This concept of correlation is at the core of many practical applications of wavelet theory.

As a mathematical tool, wavelets can be used to extract information from many different kinds of data, including – but not limited to – audio signals and images. Sets of wavelets are generally needed to analyze data fully. A set of "complementary" wavelets will decompose data without gaps or overlap so that the decomposition process is mathematically reversible. Thus, sets of complementary wavelets are useful in wavelet based compression/decompression algorithms where it is desirable to recover the original information with minimal loss.

In formal terms, this representation is a wavelet series representation of a square-integrable function with respect to either a complete, orthonormal set of basis functions, or an overcomplete set or frame of a vector space, for the Hilbert space of square integrable functions. This is accomplished through coherent states.

Table S1 MRI Acquisition Parameters for the Training and Test Datasets

| Scanner | Sequence | TR/ TE (ms) | FOV (mm) | Matrix | Sections Thickness (mm) | Section  Gap (mm) | No. of Sections | Flip Angle |
| --- | --- | --- | --- | --- | --- | --- | --- | --- |
|  |  |  |  |  |  |  |  |  |
| GE 3.0T (Discovery MR 750) | T2WI | (5000-8000)/(86-100) | (400, 300) | (320, 320) | 5 | 2.5 | 2 | 120 |
|  | DWI | (3000-7500)/(46-66) | (380, 300) | (128, 130) | 5 | 1 | 1 | 90 |
|  | HBP | (2.9-3.8)/(1.1-1.6) | (360, 420) | (256, 192) | 3 | 0 | 1 | 9-15 |
| UI 1.5T (uMR 588) | T2WI | (4500-6700)/(80-90) | (350, 350) | (320, 320) | 5 | 2 | 2 | 150 |
|  | DWI | (3085-7060)/(45-70) | (380, 300) | (128, 128) | 6 | 1 | 1 | 90 |
|  | HBP | 6.7/2.4 | (400, 260) | (400, 320) | 3 | 0 | 1 | 10-12 |
| GE 3.0T (Signa HDx) | T2WI | (3000-6000)/(67.6-85) | (350, 350) | (320, 224) | 5-6 | 2 | 0.5 | 100-145 |
|  | DWI | (7100-9230)/(66-79.6) | (400, 320) | (128, 128) | 6 | 1 | 1 | 90 |
|  | HBP | 6.8/2.4 | (380,250) | (400, 310) | 6 | 0 | 1 | 12 |

TR, repetition time; TE, echo time; ms, millisecond; FOV, field of view; No., number; mm, millimeter; DWI, diffusion-weighted imaging; T2WI, T2-weighted image; HBP, hepatobiliary phase

Table S2 The Performance of Single-sequence Models Based on Multiple VOIs in Training Cohort

| Sequences | Feature Count | AUC | Accuracy | Sensitivity | Specificity | PPV | NPV |
| --- | --- | --- | --- | --- | --- | --- | --- |
| DWI |  |  | \|  \| \| --- \| | \|  \| \| --- \| | \|  \| \| --- \| | \|  \| \| --- \| | \|  \| \| --- \| |
| VOI_tumor_ | 21 | 0.904 (95% CI: 0.882, 0.923) | 0.810 (95% CI: 0.784, 0.836) | 0.760 (95% CI: 0.718, 0.802) | 0.851 (95% CI: 0.818, 0.884) | 0.809 (95% CI: 0.769, 0.849) | 0.811 (95% CI: 0.776, 0.845) |
| VOI_3mm_ | 32 | 0.879 (95% CI: 0.855, 0.901) | 0.802 (95% CI: 0.776, 0.828) | 0.747 (95% CI: 0.703, 0.786) | 0.847 (95% CI: 0.814, 0.879) | 0.802 (95% CI: 0.763, 0.843) | 0.802 (95% CI: 0.767, 0.834) |
| VOI_5mm_ | 35 | 0.943 (95% CI: 0.926, 0.957) | 0.879 (95% CI: 0.856, 0.899) | 0.859 (95% CI: 0.823, 0.892) | 0.894 (95% CI: 0.863, 0.921) | 0.871 (95% CI: 0.837, 0.903) | 0.885 (95% CI: 0.853, 0.912) |
| VOI_10mm_ | 30 | 0.833 (95% CI: 0.807, 0.857) | 0.749 (95% CI: 0.7205, 0.7759) | 0.693 (95% CI: 0.647, 0.738) | 0.795 (95% CI: 0.759, 0.837) | 0.737 (95% CI: 0.694, 0.782) | 0.758 (95% CI: 0.722, 0.795) |
| VOI_15mm_ | 33 | 0.877 (95% CI: 0.854, 0.900) | 0.796 (95% CI: 0.770, 0.824) | 0.755 (95% CI: 0.713, 0.798) | 0.830 (95% CI: 0.795, 0.863) | 0.7860 (95% CI: 0.744, 0.828) | 0.804 (95% CI: 0.768, 0.841) |
| VOI_20mm_ | 29 | 0.611 (95% CI: 0.571, 0.651) | 0.452 (95% CI: 0.418, 0.487) | 0.971 (95% CI: 0.954, 0.986) | 0.022 (95% CI: 0.009, 0.036) | 0.451 (95% CI: 0.416, 0.488) | 0.476 (95% CI: 0.250, 0.684) |
| T2WI |  |  |  |  |  |  |  |
| VOI_tumor_ | 16 | 0.833 (95% CI: 0.8046, 0.858) | 0.772 (95% CI: 0.742, 0.802) | 0.659 (95% CI: 0.611, 0.705) | 0.866 (95% CI: 0.834, 0.897) | 0.803 (95% CI: 0.757, 0.845) | 0.754 (95% CI: 0.715, 0.791) |
| VOI_3mm_ | 14 | 0.825(95% CI: 0.798, 0.851) | 0.731 (95% CI: 0.702, 0.759) | 0.6510 (95% CI: 0.602, 0.695) | 0.797 (95% CI: 0.758, 0.835) | 0.727 (95% CI: 0.679, 0.774) | 0.734 (95% CI: 0.695, 0.771) |
| VOI_5mm_ | 17 | 0.836 (95% CI: 0.810, 0.862) | 0.726 (95% CI: 0.698, 0.755) | 0.651 (95% CI: 0.603, 0.695) | 0.789 (95% CI: 0.751, 0.826) | 0.718 (95% CI: 0.672, 0.767) | 0.732 (95% CI: 0.692, 0.767) |
| VOI_10mm_ | 15 | 0.843 (95% CI: 0.815, 0.868) | 0.756 (95% CI: 0.726, 0.784) | 0.685 (95% CI: 0.640, 0.7293) | 0.815 (95% CI: 0.780, 0.849) | 0.754 (95% CI: 0.708, 0.802) | 0.758 (95% CI: 0.718, 0.792) |
| VOI_15mm_ | 17 | 0.683 (95% CI: 0.647, 0.720) | 0.462 (95% CI: 0.428, 0.495) | 1.000 (95% CI: 1.000, 1.000) | 0.017 (95% CI: 0.007, 0.030) | 0.457 (95% CI: 0.424, 0.491) | 1.0 (95% CI: 1.000, 1.000) |
| VOI_20mm_ | 17 | 0.596 (95% CI: 0.555, 0.636) | 0.636 (95% CI: 0.605, 0.669) | 0.482 (95% CI: 0.428, 0.531) | 0.762 (95% CI: 0.725, 0.800) | 0.627 (95% CI: 0.571, 0.680) | 0.640 (95% CI: 0.603, 0.680) |
| HBP |  |  |  |  |  |  |  |
| VOI_tumor_ | 17 | 0.832 (95% CI: 0.802, 0.857) | 0.730 (95% CI: 0.699, 0.759) | 0.672 (95% CI: 0.624, 0.720) | 0.778 (95% CI: 0.741, 0.817) | 0.803 (95% CI: 0.757, 0.845) | 0.741 (95% CI: 0.700, 0.782) |
| VOI_3mm_ | 17 | 0.832 (95% CI: 0.804, 0.857) | 0.746 (95% CI: 0.717, 0.774) | 0.667 (95% CI: 0.616, 0.711) | 0.812 (95% CI: 0.777, 0.846) | 0.746 (95% CI: 0.696, 0.789) | 0.747 (95% CI: 0.708, 0.782) |
| VOI_5mm_ | 17 | 0.848 (95% CI: 0.821, 0.873) | 0.778 (95% CI: 0.751, 0.807) | 0.748 (95% CI: 0.704, 0.792) | 0.747 (95% CI: 0.704, 0.792) | 0.759 (95% CI: 0.718, 0.801) | 0.795 (95% CI: 0.754, 0.831) |
| VOI_10mm_ | 18 | 0.846 (95% CI: 0.817, 0.8709) | 0.771 (95% CI: 0.742, 0.798) | 0.747 (95% CI: 0.701, 0.788) | 0.791 (95% CI: 0.754, 0.827) | 0.747 (95% CI: 0.699, 0.790) | 0.791 (95% CI: 0.750, 0.829) |
| VOI_15mm_ | 16 | 0.800 (95% CI: 0.770, 0.829) | 0.728 (95% CI: 0.699, 0.757) | 0.635 (95% CI: 0.589, 0.690) | 0.804 (95% CI: 0.764, 0.839) | 0.728 (95% CI: 0.682, 0.775) | 0.727 (95% CI: 0.689, 0.767) |
| VOI_20mm_ | 17 | 0.810 (95% CI: 0.780, 0.837) | 0.746 (95% CI: 0.718, 0.776) | 0.656 (95% CI: 0.611, 0.703) | 0.821 (95% CI: 0.786, 0.854) | 0.752 (95% CI: 0.705, 0.795) | 0.743 (95% CI: 0.707, 0.780) |

AUC, area under the curve; PPV, positive predictive value; NPV, negative predictive value ; VOI, volume of interest; DWI, diffusion-weighted imaging; T2WI, T2-weighted image; HBP, hepatobiliary phase


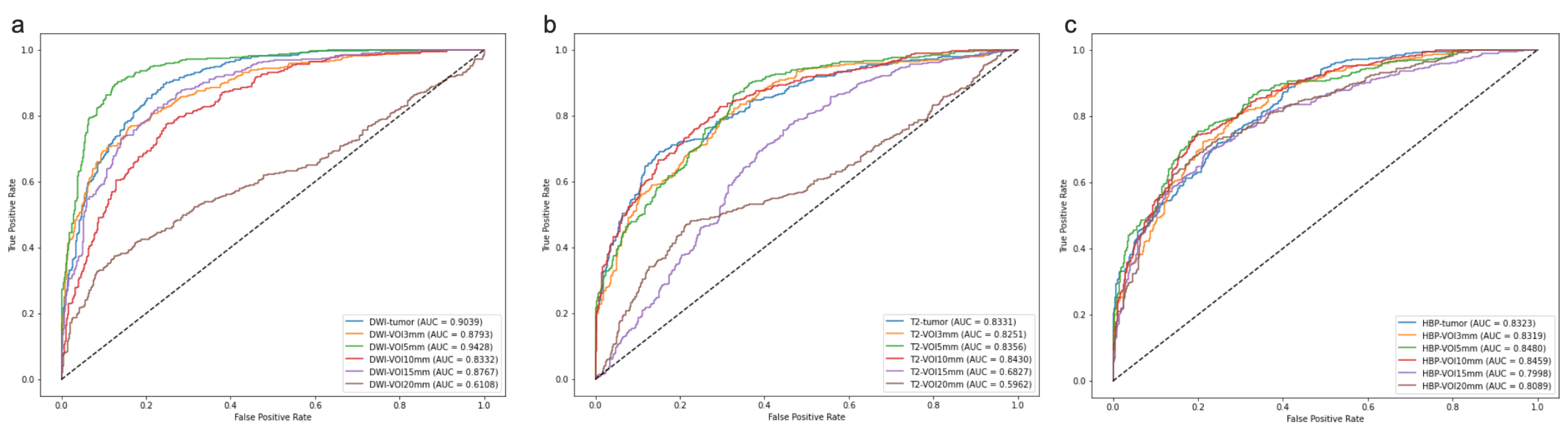


Fgiure S1 The receiver operating characteristic curves of multiple VOIs in the training cohort based on DWI (a), T2WI (b), and HBP (c) sequences.
